# Zoonotic Animal Influenza Virus and Potential Mixing Vessel Hosts

**DOI:** 10.3390/v15040980

**Published:** 2023-04-16

**Authors:** Elsayed M. Abdelwhab, Thomas C. Mettenleiter

**Affiliations:** 1 Institute of Molecular Virology and Cell Biology, Friedrich-Loeffler-Institut, Federal Research Institute for Animal Health, Südufer 10, 17493 Greifswald-Insel Riems, Germany; 2 Friedrich-Loeffler-Institut, Federal Research Institute for Animal Health, Südufer 10, 17493 Greifswald-Insel Riems, Germany

**Keywords:** pandemic, animal influenza, interspecies transmission, zoonoses, public health, avian influenza, swine influenza, mixing vessel hosts

## Abstract

Influenza viruses belong to the family *Orthomyxoviridae* with a negative-sense, single-stranded segmented RNA genome. They infect a wide range of animals, including humans. From 1918 to 2009, there were four influenza pandemics, which caused millions of casualties. Frequent spillover of animal influenza viruses to humans with or without intermediate hosts poses a serious zoonotic and pandemic threat. The current SARS-CoV-2 pandemic overshadowed the high risk raised by animal influenza viruses, but highlighted the role of wildlife as a reservoir for pandemic viruses. In this review, we summarize the occurrence of animal influenza virus in humans and describe potential mixing vessel or intermediate hosts for zoonotic influenza viruses. While several animal influenza viruses possess a high zoonotic risk (e.g., avian and swine influenza viruses), others are of low to negligible zoonotic potential (e.g., equine, canine, bat and bovine influenza viruses). Transmission can occur directly from animals, particularly poultry and swine, to humans or through reassortant viruses in “mixing vessel” hosts. To date, there are less than 3000 confirmed human infections with avian-origin viruses and less than 7000 subclinical infections documented. Likewise, only a few hundreds of confirmed human cases caused by swine influenza viruses have been reported. Pigs are the historic mixing vessel host for the generation of zoonotic influenza viruses due to the expression of both avian-type and human-type receptors. Nevertheless, there are a number of hosts which carry both types of receptors and can act as a potential mixing vessel host. High vigilance is warranted to prevent the next pandemic caused by animal influenza viruses.

## 1. Influenza Viruses

### 1.1. Classification and Structure

The virus family *Orthomyxoviridae*, a member of the Order *Articulavirales* in the Phylum *Negarnaviricota* [1], contains four genera of influenza virus, designated as Alphainfluenzavirus, Betainfluenzavirus, Deltainfluenzavirus, and Gammainfluenzavirus. For each of these four genera, only one type of species is known, and these are named Influenza A virus (IAV), Influenza B virus (IBV), Influenza C virus (ICV), and Influenza D virus (IDV) [1]. IAV can be further divided according to the host of origin into, e.g., human influenza virus (hIAV), swine influenza virus (swIAV), avian influenza virus (AIV), equine influenza virus (EIV), canine influenza virus (CIV), and bat influenza virus [2]. According to the antigenic variations of the surface glycoproteins, hemagglutinin (HA or H), and neuraminidase (NA or N), IAVs are divided into distinct 18 HA (H1-H18) and 11 NA (N1-N9) subtypes. hIAVs contain mostly H1, H2, H3 and N1, N2 subtypes, AIVs contain H1 to H16 and N1 to N9 subtypes, while H17N10 and H18N11 are so far only detected in bats [2,3]. 

IAV and IBV have a pleomorphic shape typically with a length of 80 to 120 nm. Few IAV and IBV look filamentous with variable lengths. They contain a lipid-bilayer envelope obtained from the host cell plasma membrane during virus budding. The genome is composed of eight single-stranded RNA segments with negative polarity (segments one to eight) and are named after the main encoding proteins [4]. The eight segments of IAVs encode at least ten viral proteins. Nine proteins are included in the virion: PB2, PB1, PA, HA, NP, NA, M1, M2, and NEP, while the non-structural protein NS1 is expressed only in host cells after infection. Structural proteins are differentiated into surface and internal proteins. On the viral envelope, there are the three surface proteins HA, NA, and the less abundant ion-channel matrix-2 (M2) protein. They are imbedded in the viral envelope by transmembrane domains [5,6]. Beneath the virus envelope, the nuclear export protein (NEP) is bound to the matrix-1 (M1) protein, which interacts with the C-terminal endo-domains of HA, NA, and M2. All RNA segments are connected by the polymerase subunits (polymerase basic PB2 and PB1, polymerase acidic PA) and wrapped into a nucleoprotein NP coat to form the ribonucleoprotein complex (RNP). The RNPs are the main replication units of IAV [7]. 

**Figure 1:**
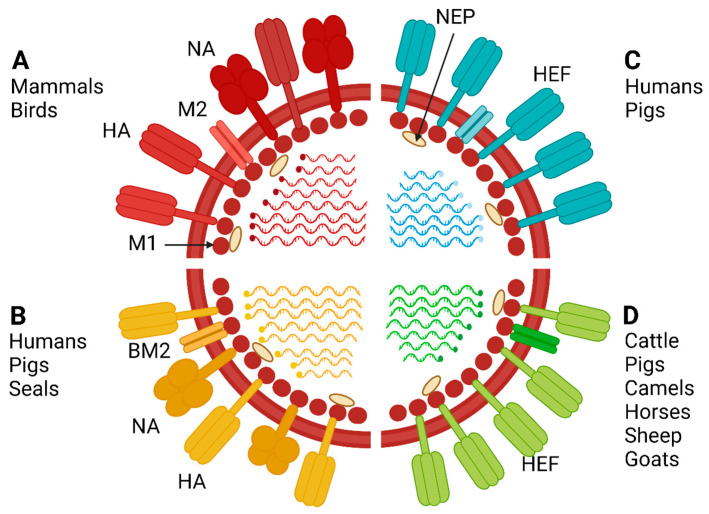
Influenza A, B, C, and D schematic structure and host range. The genome of Influenza A and B is composed of 8 gene segments, while that of influenza C and D is composed of 7 gene segments. Influenza C and D encode hemagglutinin esterase (HEF) protein, which is equivalent to the HA and NA proteins of Influenza A and B. Influenza A infects a wide range of mammals (including humans) and all bird species. The figure was created with BioRender.

IBV has a similar structure but specifies four envelope proteins HA, NA, and, instead of M2, NB and BM2. Influenza C and D viruses contain only seven gene segments. They possess one major surface glycoprotein, the hemagglutinin-esterase-fusion (HEF) protein, which corresponds functionally to the HA and NA of IAV and IBV, and one minor envelope protein, CM2, which is similar to the M2 of IAV [7,8]. Only IAV and, to a lesser extent, IDV infect a broad range of mammalian species (Figure 1). Therefore, we will focus in this review on IAV and will shortly describe the importance of IDV.

### 1.2. Virus Evolution (Shift and Drift)

Despite the relatively small genome, IAVs developed many strategies to ensure successful evolution and to expand their host spectrum. Unlike the majority of RNA viruses, IAV replication occurs in the nucleus [9]. To overcome the limited genome coding capacity, IAV encodes several non-structural proteins (e.g., NS1, PB1-F2, and PA-x) through methods such as splicing, alternative translational initiation, or frameshift. These proteins play diverse roles in, e.g., interferon antagonism, virus replication, or interspecies transmission [10,11]. Swapping gene segments (also known as reassortment) of two influenza viruses infecting the same host cell is a major evolutionary route for the generation of reassortant viruses with phenotypic properties different from their ancestors. For example, several influenza pandemics were caused by human (seasonal) influenza viruses carrying gene segments from AIV and/or swIAV. Compared to the parental human, avian and/or swine viruses, the pandemic influenza viruses had severe clinical outcomes in humans [12]. Another very important property is that IAV polymerase lacks proof-reading ability, and error-prone changes evolve de novo, generating a highly diverse quasispecies of viruses with different mutations. Some of these mutations can be deleterious for virus replication and will be eliminated from the quasispecies, while others can be fixed and confer efficient replication and perpetuation in different hosts and niches. Some mutations can result in antigenic drift, particularly after transmission to a new host or vaccination [13,14].

## 2. Host Range of (Zoonotic) Influenza A Virus

### 2.1. Birds 

#### 2.1.1. Wild Birds 

Wild birds are the natural reservoir for all subtypes of avian influenza viruses. AIV have been reported in at least 100 species in 12 out of 50 avian orders [15]. Aquatic birds and shorebirds of the orders Anseriformes (e.g., Mallard ducks) and Charadriiformes (e.g., gulls, terns) are the most common reservoirs of AIV [16,17]. Generally, wild birds have not not shown any clinical signs after infection with AIV, with a few exceptions [16,18]. The central dogma is that the replication of AIV in these wild bird species is predominantly in the intestinal tract and viruses are excreted in the fecal matter and remain infectious for weeks in the water or wet fecal matters [19]. This facilitates the fecal–oral transmission among different wild bird species and mediates the intercontinental spread of AIV through bird migration [20,21,22]. In wild birds, AIVs are generally of low pathogenicity (LP). However, the incursion of high pathogenicity (HP) AIV H5Nx into wild birds is a game-changer. Many wild bird species have succumbed to unusual high mortality and H5Nx-induced mass die-offs threaten the wildlife-ecosystem balance. Moreover, the virus can be transported over long distances by migratory birds, become enzootic in resident bird populations, exhibit high virulence in domestic birds, have zoonotic potential, and infect several mammalian species worldwide [23,24,25,26]. 

#### 2.1.2. Domestic Birds 

Poultry production is a major source for meat protein. Its drastic increase in past decades led to a change in the natural epidemiology of AIV. AIVs have been identified in all domestic and caged bird species (e.g., chickens, turkeys, waterfowls, ostrich, pigeons, quails, pet birds, gamebirds, zoo birds) [27]. While viruses of H12 to H16 subtypes are less or have not been isolated from poultry so far (except for a single historic H13N2 isolated from turkeys), viruses of H1 to H11 subtype are more frequently detected. Indeed, some AIV subtypes are enzootic in poultry in some countries, e.g., H5Nx in Asia and Africa; H6N2 in South Africa, China, and Korea; H7N3 in Mexico; H7N9 in China; and H9N2 worldwide [28,29,30]. Direct contact of poultry with wild birds or contaminated fomites are the main pathways for the primary introduction of AIV into poultry. The movement of vehicles and humans, for example, are important sources for secondary spread [30]. The transmission of some AIVs from poultry to wild birds has also been reported [31]. 

Virulence and transmissibility of AIVs in poultry vary considerably according to factors related to the virus (e.g., subtype, pathotype, route of infection, inoculation dose), host (e.g., species, breed, age) and environment (e.g., hygiene, humidity, temperature, wind). AIV infection in poultry ranges from asymptomatic to 100% morbidity and mortality [30]. All AIV subtypes in poultry are of LP and do not cause overt clinical signs or induce mild respiratory symptoms and a reduction in meat and egg production [27]. However, the transmission of LPAIV H5 and H7 to poultry may result in a transition from a LP to a HP phenotype. HPAIV H5 and H7 can lead to 100% morbidity and mortality in poultry, mainly chickens and turkeys, within a few days. Remarkably, all AIVs that are HP in ducks are also HP in chickens, but not vice versa. In ducks, the majority of HPAIV H5/H7 are avirulent in contrast to chickens and turkeys [32,33,34]. Moreover, duck species vary in their response to infection with different HPAIV, mainly H5N1 [35]. Mallard ducks are considered the major reservoir of AIV. They are usually resistant to AIV-induced morbidity and mortality. However, several H5N1 and H5N8 viruses are also highly virulent in mallards [36]. Several studies have shown that Muscovy ducks were more sensitive than Pekin ducks [35,37,38]. Furthermore, young ducklings succumbed with high morbidity and mortality after infection with some HPAIV compared to older ducks [35,39], indicating an age-dependent response. Interestingly, the virulence determinants for the transition of LP H5/H7 to HP can be different for different poultry species [40,41]. Therefore, LP H5, and H7 viruses are notifiable to the World Organization for Animal Health (WOAH), and affected flocks should be culled to prevent the transition to a HP phenotype. Conversely, the current regulations do not mandate the eradication of poultry in case of non-H5/H7 viruses [30]. The circulation of these non-notifiable low virulent viruses affects poultry production and endangers human health per se or after reassortment with other IAV. Vaccination has been used in different developing countries to limit the losses caused by LP and HP AIV [42].

### 2.2. Mammals 

#### 2.2.1. Humans

Seasonal influenza in humans is caused by subtypes H1, H2, H3 and N1 and N2, which are antigenically and genetically distinct from swIAV (reviewed in [43]). H1N1, H3N2, and H1N2 are the current predominant subtypes circulating the human population. Seasonal epidemics, mostly in colder seasons, are usually caused by H1N1 and H3N2, the latter exhibiting a more rapid antigenic drift than H1N1. The H1N2 subtype is a human/swine IAV reassortant of the circulating H1N1 and H3N2. It evolved in the early 2000s and is still circulating, although at lower rates than H1N1 and H3N2. H2N2 was circulating in humans from 1957 to 1968 and was replaced by H3N2 [44]. Direct person-to-person infection via the respiratory tract is the main route of transmission. The severity of influenza infections in humans range from asymptomatic to serious with the affection of both, the upper (URT) and lower respiratory tract (LRT) [43]. Fever, chills, headache, sore throat, myalgias, malaise, anorexia, and pneumonia are common symptoms. Immunocompromised patients and people > 65 years have a higher case fatality rate (CFR). The virus replicates mainly in the LRT and URT [45]. Small amounts of infectious virus were found in the blood, viscera, brain, and cerebrospinal fluid in only a few rare and mostly fatal cases. Virus-induced pneumonia or secondary bacterial superinfection are typically the reason for severe illness and/or fatality in patients with influenza virus infection. Strong pro-inflammatory reactions, known as a “cytokine storm”, together with high viral replication rates in the LRT, are characteristics of primary viral pneumonia [46]. Due to the seasonal spread of hIAV in cold months, viruses in the Northern and Southern hemispheres can be genetically and antigenically different. This has implications for the selection of vaccine strains, which are regularly updated to improve vaccine efficacy to protect against antigenic-drift viruses [47]. The use of antivirals targeting the neuraminidase (e.g., oseltamivir), polymerase (e.g., peramivir), or to a lesser extent, M2 (e.g., adamantane HCL) are options for controlling hIAV infections. However, the evolution of resistant variants (particularly amantadine resistant and, less frequently, oseltamivir-resistant) is a challenge in the treatment of influenza [48].

#### 2.2.2. Pigs

swIAV of the three subtypes H1N1, H1N2, and H3N2 circulate in pigs globally (reviewed in [49,50,51]), causing high economic losses in pig husbandry. The simultaneous detection of multiple swIAV subtypes is common in pigs. Pig-to-pig transmission usually occurs through close contact and possibly via contaminated objects moving between infected and uninfected pigs. swIAV cause an acute respiratory infection ranging from asymptomatic to mild fever, depression, respiratory disorders (e.g., coughing, sneezing, nasal and ocular discharges, dyspnoea), and body weight loss. Virus replication is usually restricted to the epithelial cells of the entire respiratory tract, notably the nasal mucosa, tonsils, trachea, and lungs. Virus isolation from extra-pulmonary tissues is very rare. The virus induces low mortality and recovery occurs generally within 7–10 days. Biosecurity measures and vaccination are commonly used to control IAVs in pigs. The majority of swIAV are reassortants, combining genes from swine, avian, and human viruses. This supports the main dogma that pigs can act as a “mixing vessel” between human and avian influenza viruses.

#### 2.2.3. Other Mammals

Several mammalian species succumb to sporadic infections with different influenza viruses. These spill-over events usually go unnoticed, but can sometimes be fatal, particularly when combined with bacterial or viral co-infections [52,53]. Nevertheless, and rarely, some AIVs have become endemic in different livestock and companion animals after interspecies spill-over [54]. For instance, the H3N8 EIV seems to have emerged from an AIV that spread to horses and then onto dogs. Similarly, canine H3N2 influenza viruses originated wholly from avian ancestors in the 1990s [54,55,56]. Likewise, the endemic IDV in cattle (discovered in 2011) showed a high degree of genetic similarity to the human ICV. This suggests a common ancestor for both viruses [8]. Infected horses, dogs, and cattle exhibit a mild respiratory disease similar to flu-like illness caused by hIAV and swIAV. Commercial vaccines are used in horses and dogs to mitigate the outcome of IAV infections or to limit transmission [57]. 

## 3. Zoonotic Influenza Viruses

Humans are generally partially immunized against severe influenza symptoms due to previous infections or (annual) vaccination against hIAV. However, the lack of pre-existing immunity to antigenically novel HA/NA in the human population may result in high levels of virus replication and transmission [58]. In addition, severe immune responses to the novel virus may trigger a “cytokine storm” and subsequently severe symptoms and high CFR [46]. The regular sporadic human infections with animal influenza viruses represent a continuous risk for public health. We classified these zoonotic viruses into two groups based on the available data on the frequency of animal-to-human transmission. While bovine, equine, canine, and bat influenza viruses pose a low zoonotic risk to humans, avian and swine influenza viruses pose a high zoonotic risk.

### 3.1. Animal Influenza A Viruses with High Zoonotic Potentials

#### 3.1.1. Zoonotic Avian Influenza A Virus 

##### Confirmed Human Infections

**Figure 2:**
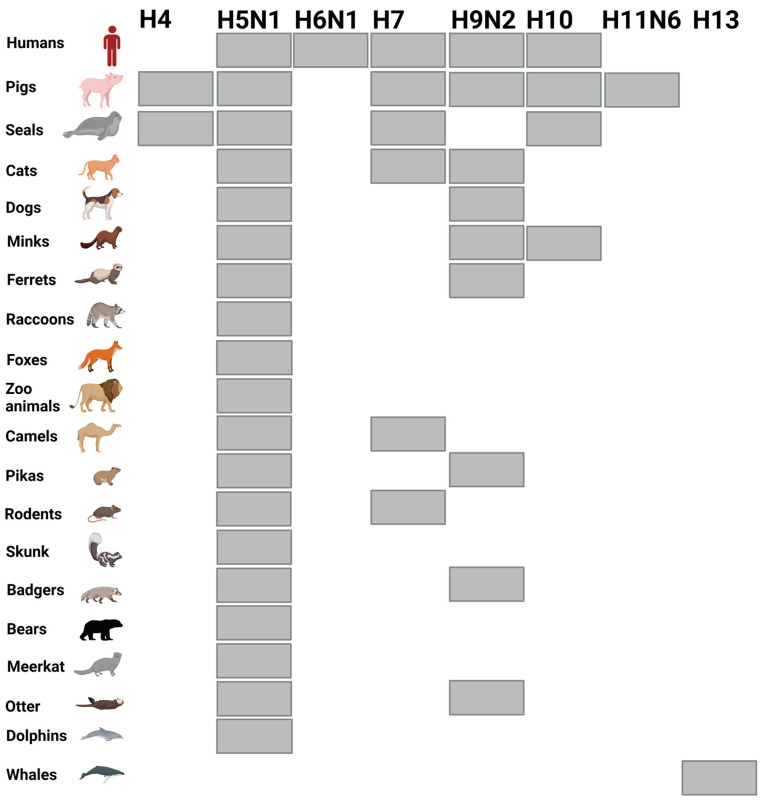
Transmission of AIVs to mammals. Grey boxes refer to the confirmed infection of humans and indicated animals with AIV subtypes written in the upper line. The figure is created with BioRender.

AIV infects a broad range of mammals (Figure 2) [59,60,61,62]. For instance, H5N1 has been naturally isolated from cats, dogs, foxes, seals, leopards, Mustelidae (minks and otters), skunks, tigers, lions, pikas, otters, polecats, porpoises, raccoons, raccoon dogs, pigs, Virginia opossums, civets, badgers, bears, dolphins, stone/beech martens, coyotes, and fishes [26,62]. H9N2 has been reported naturally in pigs, dogs, horses, minks, otters, pikas, bats, and Asian badgers [63,64,65].

In humans, a number of AIVs succeeded to cross the species barriers and establish productive infections, including H3N8, H5N1, H5N6, H5N8, H6N1, H7N2, H7N3, H7N4, H7N7, H7N9, H9N2, H10N3, H10N7, and H10N8 subtypes (Table 1). The infections ranged from asymptomatic to mild to fatal. The infections were mostly commonly acquired through direct contact with infected poultry or contaminated environment. Major sources of infection are live bird markets (LBM), backyard birds, slaughterhouses, and the culling of farmed poultry [66,67]. To a lesser extent, humans have been infected via hunting or contact with wild birds [68,69]. Cultural and occupational aspects play a major role in human infections with zoonotic influenza viruses. In some countries, the prevalence of AIVs was higher in women, children, and toddlers than in men [70,71]. Likewise, immunosuppression and chronic diseases facilitate the development of severe influenza symptoms. Aerosol transmission during slaughter, evisceration, and defeathering of live poultry are among the main sources of infection. Humans acquire infection via respiratory droplets through the nostrils or through the conjunctiva. Fine aerosols may deliver virus particles to the LRT, triggering severe illness. Virus replication is usually restricted to the respiratory tract, but extra pulmonary virus replication, including in the brain, has also been reported [72,73]. 

Flu-like illness limited to sneezing, runny nose, and fever has been reported. These mild infections are usually self-limiting. However, the number of hospitalizations can be high in the case of immunosuppressed patients or in the case of the infection with more pathogenic viruses such as H7N9 and to a lesser extent H5N1 [74,75]. Importantly, there is no correlation between high virulence in chickens and humans. For example, H7N9 in chickens was LP, causing no or only mild clinical signs, while in humans it produced severe or even fatal infections [76]. Based on the number of reported laboratory-confirmed cases, the CFR for H5 and H7 AIV in humans is relatively high (~53%; 457/868 for H5N1 and ~39%; 616/1568 for H7N9) [77,78]. It is worth noting that the pandemic H1N1 in 1918, H2N2 in 1957, and H3N2 in 1968 were most likely of avian origin, and the pandemic H1N1 in 2009 (designated hereafter as pdmH1N1) contained genes from AIV [79].

**Table 1:** Reported human infections with AIV from 1959 to 2023.

Subtype	Year of First Human Identification	Year of Last Human Identification	Number (Fatal Cases)	Country	References
H3N8	2022	2022	2	China	[80,81]
H5N1	1997	1997	18 (6)	Hong Kong	[82]
	2003	2023	868 (457)	Many *	[80,83]
	2022	2023	5	UK, USA, Spain, Ecuador	[84,85,86]
H5N6	2014	2021	83 (33)	China	[83]
H5N8	2020	2020	7	Russia	[87]
H6N1	2013	2013	1	Taiwan	[88]
H7N2	2002	2016	8	UK, USA	[88]
H7N3	2004	2012	5	Canada, Mexico, UK	[88,89]
H7N4	2018	2018	1	China	[83]
H7N7	1959	2013	96 (1)	USA, Australia, Netherlands, Italy, UK	[88,90]
H7N9	2013	2017	1568 (616)	China, Taiwan	[83]
H9N2	1998	2014	19	China, Bangladesh, Hong Kong	[65]
	2015	2022	85 (2)	China, Cambodia, Egypt	[80,83]
H10N3	2021	2022	2	China	[80]
H10N7	2004	2010	4	Egypt, Australia	[88]
H10N8	2013	2014	3 (2)	China	[88]
Total	From 1959 to 2023		2775 (1117)		

* As of 07-03-2023, from 2003 to 26 February 2023, 21 countries reported 868 confirmed H5N1-human infections with a CFR of 53% (457/868) [80,84,88]. In the table, the number of infected humans includes the number of fatal cases (between parenthesis).

##### Limited Human-to-Human Transmission of AIV

Fortunately, human-to-human transmission of AIV is still rare. Limited human-to-human transmission has been reported following infection with H5N1 and H7N9 in a few family clusters and healthcare workers in several Asian countries [91,92,93,94,95,96]. Similarly, human-to-human transmission of H7N7 from poultry workers to a few household contacts was described in the Netherlands in 2003 [97,98].

##### Subclinical Infection with AIV Is More Prevalent Than Laboratory Confirmed Infections

Subclinical AIV infection, as determined by the presence of antibodies, has been described in a number of serosurveys from different countries, yielding approximately 6639 positive cases out of 138,730 individuals tested (Table 2). Earlier systematic and meta-analysis reviews have been consulted [99,100,101,102,103,104,105,106]. The majority of these cases were detected in exposed individuals in LBM, backyards, or commercial farms and in healthcare workers. However, in some cases, antibodies have been detected in non-occupationally non-exposed individuals [96]. Interestingly, some of the sub-clinically infected individuals had antibodies against seasonal hIAV and AIV [107]. The prevalence of anti-IAV antibodies in humans has been related to several factors, including sampling time, testing method, gender, and other demographic factors [104]. Some studies showed antibodies in humans against AIV H1–H13 subtypes [103,108,109,110,111,112,113,114,115,116], while most of the studies described antibodies against H5N1, H7N9, and H9N2 (Table 2), probably because these are the most widespread AIVs in poultry. There is a special attention to H9N2 virus. Serological evidence for this virus in humans has been reported from Asia (China, Cambodia, Thailand, India, Mongolia, Pakistan, Iran, Lebanon), Africa (Egypt, Nigeria), Europe (Romania), and North America (USA) [102]. In a recent meta-analysis of 45 studies conducted in China from the 1990s to 2018, including a total of 59,590 patients, the overall H9N2 seroprevalence was estimated to be 5.56% (i.e., approximately 3313 infections) [104].

#### 3.1.2. Zoonotic Swine Influenza A Virus (swIAV)

Human infection with swIAV occurs through close contact between pigs and humans, particularly in pig holdings or slaughterhouses. Interestingly, pig-to-human IAV transmission has been regularly reported, but the number of human infections is lower than for AIV. From 1959 to 2014, only 396 swIAV-confirmed human infections were reported worldwide [89]. From 2010 to 2021, fewer than 700 confirmed cases were reported worldwide, with the majority occurring in young individuals or immunocompromised patients [51]. However, several studies have shown subclinical infections in farm workers and abattoir workers ranging from 15% to 40% [103]. In contrast to AIV H5/H7, the CFR in humans infected with swIAV from 1959 to 2005 is low (up to 14%) [165]. pdmH1N1 possessed gene segments from swIAV in addition to segments from avian and human IAV [79,166,167]. The anthropozoonotic transmission of seasonal and pandemic hIAV to pigs resulted in the establishment of a long-term reservoir in pigs for zoonotic IAVs [168,169]. Of note, several AIVs, including H5, H7, and H9 viruses, have been reported in pigs [51]. Transmission of swIAV to poultry, mainly turkeys, has also been reported [170,171,172].

### 3.2. Animal Influenza A Viruses with Low Zoonotic Potential

#### 3.2.1. Bovine Influenza D Virus

IDV was detected in pigs in the USA in 2011, although cattle are the primary reservoir [173]. In addition to North America, IDV has been isolated in Europe, Asia, Africa, and South America [174,175]. IDV has the ability to expand its mammalian-host spectrum to small ruminants (sheep, goats), horses, and camelids under natural conditions, and infects mice, ferrets, and guinea pigs under experimental conditions [176]. In addition, IDV RNA was detected in the nasal wash of a pig farm worker in Malaysia in 2017 [177]. Recent studies have shown the presence of anti-IDV-antibodies in up to 46% of serologically tested individuals in Italy, the USA, and Canada [178,179], and the virus was able to replicate in human airway cell culture in vitro [180]. Therefore, special attention should be given to this virus.

#### 3.2.2. Equine Influenza A Virus (EIV) 

Although the etiology is unknown, the first well-documented influenza-like epizootic in horses was reported in the USA in 1872, but earlier outbreaks in equids have likely occurred [181]. Since the 1930s, only two main subtypes of EIVs have been identified in diseased horses: H7N7 (aka A/equi-1) and H3N8 (aka A/equi-2). H7N7 viruses have not been isolated since the late 1970s, while H3N8 viruses continue to cause sporadic outbreaks in horses worldwide [53,182,183]. Other IAV subtypes have been rarely reported in horses, such as H1N8, H5N1, H7N1, and H9N2 [182]. There are few reports of limited transmissions of H3N8 to other mammals, i.e., dogs, cats, pigs, and camels [184,185,186,187]. Isolation of equine H3N8 in humans has never been confirmed [53,183,185]. However, serological evidence for equine–human transmission has been obtained in the Ukraine in 1959, in Canada 1963, the USA in the 1960s, 2005, and 2015, the UK in 1965, the Netherlands in 1966, Mongolia in 2008–2013, and Australia in 2014, indicating subclinical infection [155,182]. Seroconversion and mild illness (e.g., fever, flu-like illness) have been described in volunteers after nasal or oropharyngeal infection with EIV H3N8 in the USA in the 1960s in three independent challenge studies [188,189,190]. 

#### 3.2.3. Canine Influenza A Virus 

Generally, dogs have not been considered a natural host for IAVs. However, since the 2000s, two major subtypes, H3N8 and H3N2, have been isolated from or become enzootic in dogs in some countries. The first outbreak of H3N8 CIV, closely related to equine H3N8, was reported in 2002 in English foxhounds in the UK [191]. Thereafter, similar outbreaks caused by equine H3N8 were reported in different regions of the USA and in Australia [192,193,194]. H3N2 CIV of avian origin was isolated from dogs in 2004–2005 in China and South Korea, and spread to the USA in 2015 [56,194]. Interestingly, dogs naturally transmitted H3N2 to cats [195]. Experimental infections have shown that H3N2 CIV infected a wide range of mammals, including ferrets, guinea pigs, and cats, but not pigs [196,197]. There is no strong evidence of the transmission of H3N2 or H3N8 CIV to humans, and the risk of human infection is considered low [198]. 

#### 3.2.4. Bat Influenza A Virus 

In 2009–2011, two new IAVs were isolated from bats in Bolivia and Guatemala [199]. The H18N11 viruses did not cause any disease in ferrets (the standard animal model for assessing the zoonotic potential of influenza viruses) and were not transmitted between them. However, there is some evidence that the new bat-origin influenza A virus may be able to enter and replicate in human cells [200,201,202,203,204,205]. Therefore, the risk of potential zoonotic spillover of the various bat IAVs should not be neglected.

## 4. Potential “Mixing Vessel” Hosts

**Figure 3:**
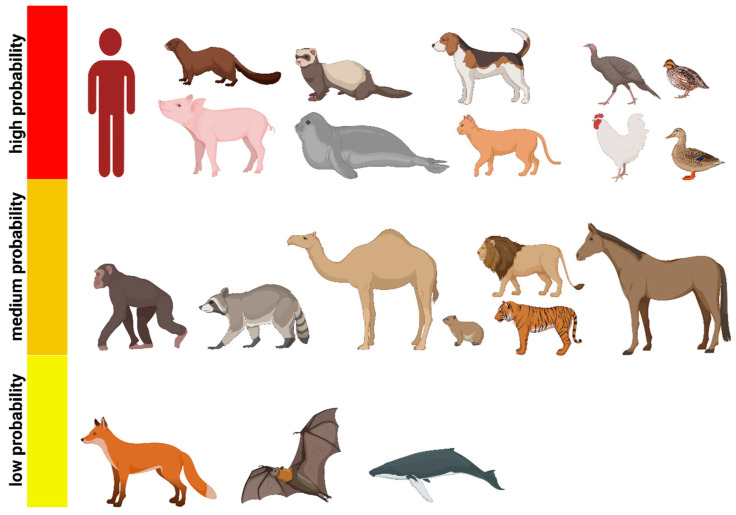
Potential “mixing vessel” hosts for the generation of zoonotic animal influenza viruses. Potential mixing vessel hosts according to the frequency of infection, close contact with humans, the high number of populations, and the distribution of avian- and human-type receptors. Humans, pigs, minks, ferrets, seals, dogs, cats, and birds, particularly turkeys, chickens, quails, and ducks, are the “high probability” mixing vessel hosts; “medium probability” mixing vessel hosts are non-human primates, raccoons, camels, pikas, zoo animals, including tigers and lions, and horses. The “low probability” hosts for the generation of zoonotic animal IAV are foxes, bats, and whales.

Mixing vessel hosts are those in which co-infection of two (or more) IAVs can occur simultaneously, leading to the potential for reassortment and generation of new IAV genotypes/phenotypes. They act as intermediate hosts for the spread of IAV between/to mammals, including humans. Although several host factors are incriminated in the ability of animal influenza to replicate in human cells, virus receptors on the cells are a major determinant of host susceptibility to influenza viruses and thus play an important role in infection and virulence. Sialic acid (SA) α-linked at C2 to galactose of a cellular glycoprotein or glycolipid is the most common receptor for influenza viruses. hIAV typically prefers binding to an α2,6-linked SA (galactose C6, designated hereafter as α2-6-SA) and avian IAV to an α2,3-linked (galactose C3, α2,3-SA) [206]. α2,6-SA in the respiratory tract is commonly referred to as the “human receptor” and α2,3-SA is found in the intestinal tract of birds and is referred to as the “avian receptor”. It was originally thought that humans exclusively express α2,6-SA, birds only express α2,3-SA, and pigs have both avian and human receptor types. Therefore, they play a role as a mixing vessel for the generation of different avian and human reassortants. New studies have changed this paradigm of species and tissue distribution of SA. Many mammalian and avian species possess both types of SA receptors with variable abundance, are susceptible to hIAV and AIV infection, and can play a role as mixing vessels, similar to pigs (Figure 3). As different methods have been used to identify SA receptors in understudied species, a direct comparison is not possible. Here, we summarized potential “mixing vessel” based on the distribution of avian and human SA receptors, the number of animal-to-human IAV transmission events, the number of IAV subtypes, the number of animal populations, and the direct and long contact with humans, and the severity of the disease (not dead end hosts) to “high probability”, including humans, pigs, minks, ferrets, seals, dogs, cats, and birds, particularly turkeys, chickens, quails, and ducks; “medium probability” mixing vessel hosts are non-human primates, raccoons, camels, pikas, horses, and zoo animals, including tigers and lions. The “low probability” hosts are foxes, bats, and whales (Figure 3).

### 4.1. Humans

**Figure 4:**
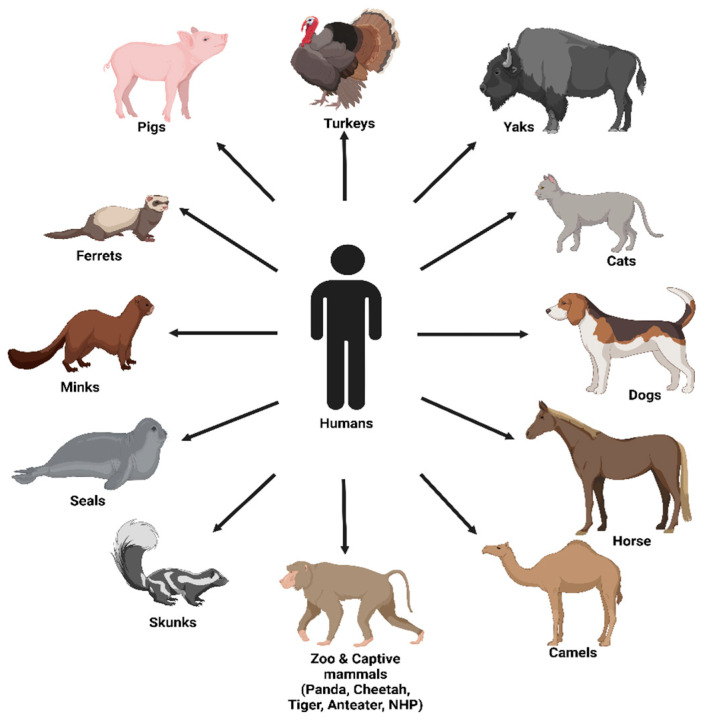
Transmission of human influenza viruses to animals (reverse zoonoses).

As shown in Table 1 and Table 2, several AIVs can infect humans directly without the need for an intermediate host. Volunteers challenged with different AIV H4N8, H6N1, and H10N7 shed virus and developed mild clinical symptoms [207]. Similarly, without prior adaptation to mammalian hosts, AIVs can bind to α2,6-SA, even more than binding to avian α2,3-SA [208,209,210,211]. hIAVs have been detected naturally in turkeys and a wide-range of mammals, including pigs, ferrets, minks, seals, dogs, cats, horses, yaks, skunks, and captive and zoo mammals (Figure 4) [51,168,212]. Several studies have shown that the human respiratory tract contains α2,3-SA (avian receptors) [213]. Interestingly, mixtures of α2,3-SA and α2,6-SA were found in the human lung and bronchus, and the expression of α2,3-SA in the bronchus was more abundant than α2,6-SA, particularly in pediatric bronchus compared to the adult bronchus [213,214]. A recent study showed that both α2,3-SA and α2,6-SA were detected in adult human alveolar N-glycans with a higher molar ratio of α2,3-SA to α2,6-SA [215]. In the human nasal cavity, both α2,3-SA and α2,6-SA receptors have been detected on ciliated epithelial cells and mucus-secreting goblet cells [214,216,217]. α2,3-SA has also been found in the colon epithelium, on the vascular endothelial cells, and on inflammatory cells [218]. Therefore, humans can act as a mixing vessel host.

### 4.2. Pigs

Pigs are the historical and best known mixing vessel for the generation of reassortant human–swine–avian influenza viruses. pdmH1N1 originated from swIAV and AIV after reassortment with hIAV. However, solid evidence on the role of pigs as a mixing vessel host for other pandemic influenza viruses is largely lacking [166,219]. Pigs are known to have both avian-type and human-type receptors and to be infected with avian and human influenza viruses. The distribution of both SA receptors in the pig respiratory tract was similar to that in the human respiratory tract [220]. The distribution of α2,6-SA in the upper airways (trachea and bronchus) was higher than α2,3-SA and both receptors were equally expressed in the lower airways (bronchiole and alveolar region) [220,221,222]. The lamina propria of the airway mucosa was dominated by α2,3-SA [220]. Another study showed that α2,6-SA was expressed on the epithelial cells along the whole respiratory tract, whereas smaller amounts of α2,3-SA were found in bronchioles and alveoli [221]. Moreover, both receptors have also been found in the liver, kidney, spleen, heart, skeletal muscle, cerebrum, small intestine, and colon [220].

### 4.3. Ferrets

Ferrets are the standard model for studying the zoonotic potential, virulence, transmission, pathogenesis, and vaccine efficacy of influenza viruses. Pet and colony ferrets have been naturally infected with swine H1N1 [223], swine H3N2 [224], pdmH1N1 [225,226,227], and avian H5N1 AIV (Van Borm et al., unpublished). The infection was mostly asymptomatic or mild and only rarely fatal [228] (Van Borm et al., unpublished). Experiments using AIV from wild and domestic birds showed efficient replication and transmissibility by direct contact and aerosol routes in ferrets without prior adaptation [229,230,231,232,233]. Several in vivo studies have shown that ferrets generate reassortant viruses after co-infection with two different hIAVs [234,235], even though the reassortment rate may vary according to, e.g., virus strain, inoculation route, infection dose, and time post-infection [12]. Interestingly, reassortment was found to be less frequent in swine than in ferrets in vivo [236]. Similar studies in ferrets found that reassortment of H5N1 AIV and H3N2 or H1N1 human viruses readily occurred in vivo [237,238]. Compared to the LRT, more reassortants were detected in the ferret nasal tract [234,236,237,238], where the airborne transmission of IAV occurs [234]. Several studies have shown that the distribution of SA receptors in the ferret respiratory tract is much more similar to the human airway than to the pig or mouse airway [239,240,241]. Both avian and human-type receptors are expressed in the alveoli. Abundant amounts of α2,6-SA have been detected in ciliated cells and submucosal glands of the ferret trachea and bronchi, whereas α2,3-SA is present in the lamina propria [239,240]. The role of ferrets as a potential mixing vessel or intermediary host for zoonotic influenza is well justified. 

### 4.4. Minks

Similar to ferrets, minks are members of the *Mustelidae* family. In contrast to ferrets, however, mink could not be domesticated as pets, but are kept in large numbers for the production of fur. The mink industry for fur production is growing in Asia, Europe, and America [242]. Farmed minks eat raw poultry and pork by-products, and they have direct and indirect contact with wild birds, pigs, and farm workers [243,244,245,246,247,248], making them a perfect mixing vessel host [249]. Several studies have shown that farmed minks were naturally or experimentally infected with various avian, human, equine, and swine IAVs with clinical signs ranging from asymptomatic to severe. They are able to transmit virus from mink-to-mink via direct contact or aerosol [249,250,251,252]. AIV H10N4 of wild-bird origin was isolated during respiratory epizootics in farmed minks in Sweden in 1984 [243,253,254,255,256,257,258]. Several mutations were observed in the HA, particularly in the receptor-binding domain, which were thought to be responsible for efficient multi-cycle replication and transmission of H10N4 virus in minks [243,253,259,260]. Interestingly, this H10N4 virus of mink origin was not able to replicate in chickens [259], suggesting progressive adaptation to mammals. Several H9N2 viruses were isolated from minks in different territories in China from 2013 to 2020, indicating a high prevalence rate in asymptomatically infected farmed minks [244,261,262]. Compared to the avian ancestors, a Chinese mink-derived H9N2 has acquired HA mutations in or adjacent to the receptor-binding domain, which is known to enhance AIV adaptation to mammals [244]. Recently, several AIVs have been isolated from clinically healthy or sick minks, including H5N1 in Spain, China, Sweden [248,262,263,264], and H5N6 in China [249]. Mink have also been infected with porcine triple reassortant H3N2 in Canada in 2007 [247], porcine H1N1 in China in 2017 [246], and porcine H1N2 in the Midwest United States in 2010 [245]. An outbreak of respiratory disease in farmed American mink caused by the pdmH1N1 was described in Norway in 2011 [265] and in the USA in 2019 [266]. Interestingly, a novel H3N2 reassortant carrying gene segments from swine H3N2 and pdmH1N1 viruses was isolated from minks in Canada in 2010 [267]. Furthermore, there is evidence of human-to-mink transmission of hIAV and co-infections with AIV. A serological surveillance conducted in 2016–2019 in 34 mink farms in China revealed that minks were commonly infected with human (H3N2 and pdmH1N1) and avian (H7N9, H5N6, and H9N2) IAVs [249]. Experimental infections have shown that minks are susceptible to human (H3N2 and pdmH1N1) and avian (H7N9, H5N6, and H9N2) IAVs and virus excretion was determined in infected minks [244,249]. In the mink respiratory tract, both receptors were found in the trachea, bronchiole, and alveoli; however, SA α2,6-Gal was more predominant [244]. Both receptors were also found in the cardiac muscles, mesenteric lymph node, and different cells in the intestine [244]. Therefore, minks can be a perfect host for the generation of zoonotic IAVs.

### 4.5. Seals

While extensive human contact with seals is difficult to imagine, seals in rehabilitation centers, parks, zoos, etc., actually do come into close contact with humans. Frequent infection of seals with human seasonal and pandemic influenza viruses has been reported [268,269,270,271] (Table 3). Importantly, seals have succumbed to morbidity and mortality after infection with different AIVs, including H4N5, H10N4, and H10N7 [272,273,274]. Seal-to-seal transmission has been observed in H5N1 infection in USA [275] and probably in an H10N7 outbreak in seals in Europe [272]. Conversely, the transmission of seal-H7N7 to humans developing conjunctivitis has been reported in 1979, in Massachusetts, USA [276]. Some seal viruses exhibited increased virulence in mice, rats, ferrets, and pigs without causing disease in experimental birds [277,278]. The distribution of SA in seals revealed the co-existence of α2,3-SA and α2,6-SA receptors in the respiratory tract [279]. α2,6-SA expression was predominant on bronchiole and alveolar epithelial cells and on endothelial cells, while the expression of α2,3-SA was scarce and limited to bronchiole luminal and alveolar epithelia [279]. Moreover, there is evidence that some AIVs displayed dual or increasing affinity to human-type receptors after acquiring de novo HA mutations in seals [280,281,282]. Likewise, mutations in the polymerase linked to mammal adaptation have also been observed after the infections of seals [274,279]. Therefore, the risk posed by seals to generate human-adapted AIVs should not be underestimated. 

### 4.6. Dogs

Although it is difficult to have an accurate estimation of the global dog population, the number of domesticated dogs is estimated to be approximately 900 million [193]. Dogs as companion animals are in close contact to humans. In addition to the established lineages of canine H3N2 and H3N8 influenza viruses, the isolation of different human seasonal and pandemic H1N1 as well as human H3N2 in addition to a number of AIVs (e.g., H5N1, H5N2, H6N1, H7N9, H9N2, and H10N8) in dogs have been described in the last few decades [193,294,295,296]. The isolation of different CIV H3N2 reassortants carrying gene segments from pdmH1N1 has been reported from dogs in South Korea [297,298]. In 2022, an AIV H3N8 infected a human in China. The viral RNA was detected in the nasopharyngeal swab of an apparently healthy dog in the patient’s house [299]. Dogs inoculated with human H3N2 viruses displayed no clinical signs, although virus shedding from the throat and seroconversion were evident [300]. Dogs possessed antibodies against hIAV, H3N2 and H1N1, and AIV (e.g., H5N1 and H9N2) as shown in several serological surveys in different countries [193,295,301,302,303]. It is worth mentioning that dogs carry both α2,3-SA and α2,6-SA receptors in their respiratory tract, particularly in goblet cells and sub-epithelial regions of nasal mucosa and trachea [304]. Both receptors were also detected in the large intestine, although α2,3-SA receptors were more abundant [304]. Given the high susceptibility of dogs to animal and human IAVs and their close contact to humans, the potential of dogs as a mixing vessel could be considered as high.

### 4.7. Cats

Similar to dogs, cats are one of the most common companion animals and are in close contact to humans. The global population of domestic cats was estimated to be about 600 million [193]. Unlike dogs, IAVs have been sporadically reported in cats. However, cats are naturally susceptible to a number of IAVs, including human seasonal and pandemic influenza viruses as well AIV H5N1, H5N6, H7N2, and H9N2 [192,305,306,307,308]. Cats inoculated with a human H3N2 virus showed no clinical signs; however, virus excretion from the throat and seroconversion were detected [300]. Several serological surveys worldwide have shown that cats have antibodies against hIAV H3N2 and H1N1 [193,295,301,302]. Cat-to-human transmission of AIV H7N2 has been reported in a veterinarian and in an animal shelter worker in the USA in 2016 [309,310]. Cats express both avian-type and human-type receptors in the respiratory tract, including the ciliated pseudostratified columnar epithelial cells and goblet cells in the trachea and alveoli epithelial cells [311,312]. Therefore, cats as pets who roam freely among birds and humans and are susceptible to avian and human influenza viruses must be considered as a potential mixing vessel for zoonotic IAV.

### 4.8. Non-Human Primates (NHPs)

There are many contexts in which humans may come into contact with NHPs, including urban settings, temples, pet NHPs, monkey performances, ecotourism, and bushmeat hunting. Under experimental conditions, NHPs are used as models to study IAV infection, pathogenesis, and immunology [313,314]. However, a number of NHPs have been infected with hIAV under natural conditions in different Asian countries [315,316,317]. Likewise, baboons in Kenya have been infected with human-like H1 and H3 viruses, most likely due to human-to-animal transmission [318]. Moreover, serological surveillance indicated the presence of antibodies to seasonal and pandemic hIAV H1, H2, and H3 in different monkeys, macaques, chimpanzees, gorillas, and orangutans in Africa, Asia, and Europe [319,320]. Studies have shown that some NHPs, including chimpanzees, gorillas, and orangutans, express an abundant amount of α2,6-SA in goblet cells but lack their expression on the epithelial cells of the trachea and the lung [218,321]. African green monkeys have a similar pattern to that of humans for the distribution of α2,3-SA and α2,6-SA receptors in the respiratory tract [322].

### 4.9. Raccoons (Procyon Lotor)

Raccoons belong to the *Carnivora* and are usually kept as pets or roam freely [323]. Raccoons were found infected with H5N1 in the USA in 2022 [26] and antibodies were detected in feral raccoons in Japan during 2005–2009 [323]. Several studies have shown that raccoons can be symptomatic or asymptomatic carriers of several AIVs or hIAVs, according to serosurveys and experimental infections. Some viruses were shed for several days and spread to other raccoons by aerosol. In some cases, raccoons have been infected simultaneously with several subtypes of IAVs [324,325,326,327,328,329]. Raccoons express both α2,3-SA and α2,6-SA receptors in the respiratory tract. α2,6-SA is predominant in the upper trachea epithelium and is equally expressed to α2,3-SA in the lungs [328].

### 4.10. Camels

Camels have been infected with equine H3N8 in Mongolia in 2012 [185], human H1N1 in Mongolia in 1978–1983 [330,331], pdmH1N1 in Nigeria in 2015–2017 [332], and avian H7N9 in China in 2020 [333]. Human/swine-like H1N1 was detected in camels imported from Djibouti and Sudan into Saudi Arabia in 2017–2018 [334]. After experimental infection with human H1N1, camels developed flu-like illness and excreted viruses between 3 and 6 dpi [330]. In 2013–2014, zoo camels in Thailand showed antibodies against the pdmH1N1 [335]. Antibodies against pdmH1N1 and H3N2 were also detected in camels in Nigeria in 2015–2017 [332]. Several serosurvey studies in camels in African countries confirmed the presence of antibodies against influenza A, B, C, and D [336,337,338,339,340]. α2,3-SA are abundant in the camel nasal respiratory epithelium and in the sub-epithelial regions, in the secretory goblet cells of the nasal epithelium, and in alveolar epithelial cells [341]. Camel erythrocytes carry high amounts of sialic acid [342]. No information is available on the expression patterns of α2,6-SA receptors in the camel respiratory tract, although both α2,3-SA and α2,6-SA have been detected in camel serum samples [343], the oviduct epithelium [344], and spermatozoa [345].

### 4.11. Plateau Pika (Ochotona Curzoniae) 

The pika is a small herbivorous rabbit-like mammal and a natural resident of the Qinghai–Tibetan Plateau. Pikas have been naturally infected with AIV (e.g., H5N1, H7N2, H9N2) [346,347,348,349] and up to 32% and 13.4% seroconversion rates against H9N2 and H5N1, respectively, have been reported in wild pikas in China [348,349]. Experimental infections showed that pikas are susceptible to hIAV H1N1 and H3N2 and AIV H5N1 [350]. Lectin staining indicated that α2,6-SA are widely expressed in the lung, kidney, liver, spleen, duodenum, ileum, rectum, and heart, whereas α2,3-SA receptors are strongly expressed in the trachea and lung [350]. Therefore, the pika may play a role as an intermediate host for the generation of zoonotic IAVs.

### 4.12. Foxes

Several studies have shown that foxes (wild or captive) are susceptible to AIVs (e.g., H9N2, H5N1) under natural and experimental conditions [26,351,352,353,354,355]. Human-adaptation markers developed after the infection of red foxes with H5N1 [353]. Studies showed the co-expression of both α2,6-SA and α2,3-SA receptors, including in the respiratory tract [355,356]. Foxes are known to feed frequently on dead birds and to prey on small animals and poultry. They play an important role in the transmission of some viral and parasitic diseases to humans [357,358]. There is therefore a risk of direct transmission of IAV from foxes to humans.

### 4.13. Bats

There exists a large number of diverse bat species. They are the natural reservoir for several zoonotic viruses. Recently, two distinct H17N10 and H18N11 IAV were detected in the yellow-shouldered bat (*Sturnira lilium*) and a fruit-eating bat (*Artibeus planirostris*) in Guatemala and Bolivia, respectively [199]. Moreover, H9N2 viruses, closely related to AIV H9N2, have also been isolated from Egyptian fruit bats (*Rousettus aegyptiacus*) in Egypt [359], and H9-antibodies were detected in 30% of straw-colored fruit bats (*Eidolon helvum*) sampled in 2009/10 in Ghana [360]. In contrast to H17N10 and H18N11, which recognize MHCII as a receptor [199], the avian-like bat H9N2 was able to bind to α2,3-SA at higher levels than to human-like α2,6-SA receptors [359]. H18N11 virus was able to infect mice and ferrets without causing any signs of disease [203]. However, infection of mammalian cell lines and animal models with H18N11 virus revealed that this virus can acquire mammal-adapting mutations that may increase its zoonotic potential [201]. A study demonstrated the SA expression in some bat species with a predominant expression of α2,3-SA in the trachea and α2,6-SA receptors in the trachea, bronchi, and lung. Both receptors were expressed in the intestine [361]. The current data suggest that bat IAVs pose a low zoonotic potential and that bats are less likely to be an intermediate for reassortment of AIV, swIAV, and hIAV.

### 4.14. Horses

Currently, there are ~60 million kept horses worldwide [362]. They are in close contact with humans. No confirmed virus isolation of EIVs from humans has been reported so far, but subclinical infections were evident by testing serum samples from humans [155,182]. Moreover, IAV subtypes, e.g., H1N8, H5N1, H7N1, and H9N2 strains, have been detected in horses, although rarely [182]. There are few reports with regard to the limited transmission of H3N8 to other mammals, i.e., dogs, cats, pigs, and camels [184,185,186,187]. Both α2,3-SA and α2,6-SA receptors were found in the respiratory tract of horses from nasal mucosa, trachea, and bronchus [363,364]. α2,3-SA was predominantly expressed on the surface of ciliated epithelial cells, whereas α2,6-SA was confined to the goblet cells [363]. However, another study showed the lack of α2,6-SA in the trachea of horses and found that horses express mainly α2,3-SA [365]. Equine IAVs are highly adapted to α2,3-SA receptors, but few mutations in the receptor-binding domain of equine IAV could facilitate the infection of other hosts (e.g., dogs, poultry) [366,367].

### 4.15. Other Mammals

There are several reports that zoo animals (e.g., tigers, leopards, lions) were infected with AIV [368]. Tigers possess both avian-type and human-type receptors in the respiratory tract [312]. Guinea pigs, hamsters, and mice possess both receptors in the respiratory tract and they are susceptible to infection and the generation of reassortant viruses with a wide-range of human and animal IAVs [369,370,371,372,373,374]. However, we did not find data on natural infection with IAV, although antibodies against IAVs were detected in guinea pigs raised as livestock in Ecuador [375]. Whales have also been found to be infected with IAV [376,377,378,379]. Mathematical models predicted that AIV transmission by whales via faecal matter along the Atlantic Coast was several times greater than that by migratory birds [380]. No information is available so far on the distribution of α2,3-SA and α2,6-SA in these giant marine mammals.

### 4.16. Birds

Poultry species or even breeds vary in the distribution of influenza virus receptors in different organs. All four major influenza pandemics were triggered by AIV either via reassortment in humans, or other yet to be identified, intermediary mammal hosts [166]. During the last three decades, frequent direct transmission of AIV from birds to humans indicate that no intermediary host is required. Moreover, birds display both avian and human-type receptors and therefore adaptation of AIV to humans can occur in bird species before transmission to humans (or other mammals).

#### 4.16.1. Chickens 

Worldwide production of chickens is estimated at 25.8 billion [362]. Chickens are infected with a wide range of AIVs [30]. In chickens, α2,3-SA is expressed in the nasal cavity [381]. Surprisingly, most studies showed that α2,6-SA dominates in the trachea of chickens over α2,3-SA [382,383,384,385,386]. Conversely, few studies reported that the epithelial cells in chicken trachea carry more α2,3-SA than α2,6-SA [387,388]. Likewise, α2,6-SA is expressed more than α2,3-SA in the lungs of chickens [385,386,389]. Both α2,3-SA and α2,6-SA are present on epithelial cells in chicken intestine as well as in kidneys and the esophagus [384,385,388,390]. Studies have shown that receptor distribution in chickens is more similar to the spectrum of receptors in the respiratory epithelia of African green monkeys than to that in the epithelial tissues of ducks [382]. Interestingly, different chicken breeds vary in the distribution of SA. For instance, white leghorn (WL) chickens and silky fowl possessed both α2,3-SA and α2,6-SA receptors in the lung and gastrointestinal (GIT) [391]. In trachea, WL had both receptors where silky fowl did not express α2,6-SA in the mucosa and lamina propria of the trachea [391]. In the GIT, both receptors were expressed in WL and silky fowl in the mucosal epithelial cells, glandular cells, and cells in the lamina propria of the gizzard-proventriculus and intestines of both breeds. In the cecum of silky fowl, the amount of cells carrying α2,3-SA and α2,6-SA were significantly lower than those in the cecum of WL [391]. In the reproductive tract of hens, except for infundibulum, both types of receptors exist particularly in the magnum, isthmus, uterus, and vagina, where the α2,3-SA was more abundant than the α2,6-SA, particularly in the columnar epithelium cells [392]. 

#### 4.16.2. Turkeys

There are about 300 million domestic turkeys worldwide [362]. Turkeys have been found to be naturally susceptible to pdmH1N1, triple reassortant H3N2 viruses, and all AIVs. They are considered a bridging host for the adaptation of wild-bird AIV to infect poultry [393]. In turkeys, generally, more avian-type receptors are expressed, but human-type receptors have increased by age [381,394]. In the nasal cavity, lung, kidney, esophagus, and intestine, both receptors were found, whereas in the trachea only an avian-type receptor was found [381,388].

#### 4.16.3. Guinea Fowls

Guinea fowls are one of the minor poultry species that are typically raised for meat. They are highly susceptible to different AIVs [395]. The lungs and trachea of guinea fowls showed a significant amount of both SA receptors, while in the large intestine only α2,3-SA was observed [394].

#### 4.16.4. Quails

Similar to Guinea fowls, quails are a minor poultry species farmed for meat and egg consumption. They are susceptible to avian, human, and swine IAVs [395]. Quails have been shown to possess both avian and human-type receptors. In the trachea, α2,6-SA is predominant than α2,3-SA. It is predominantly on the surface of ciliated cells and α2,3-SA is primarily in non-ciliated cells. In the colon, both types of receptors were found on epithelial cells as well as in crypts [387,389,390]. 

#### 4.16.5. Pheasants

Pheasants are a minor poultry species, mainly kept for meat or hunting [395]. They are highly susceptible to almost all AIV subtypes and can excrete the virus for 45 days post-infection [396]. Both types of receptors are abundant throughout the respiratory and intestinal tracts and a high expression of α2,6-SA was reported in the lungs [381,389]. 

#### 4.16.6. Ducks and Geese

There are about 1.1 billion and 370 million domestic ducks and geese worldwide, respectively [362]. Ducks and geese belong to the Anseriformes, the primary reservoir of all AIVs [395], and they are a source for zoonotic AIV [397,398]. In ducks, both types of receptors were found in the kidneys, esophagus, trachea, bronchi, and/or alveoli of Pekin and mallard ducks [381,384,388], although at lower levels than in chickens [389]. In geese, α2,3-SA was expressed throughout the respiratory tract with very low levels of α2,6-SA only in the colon [394]. 

#### 4.16.7. Pigeons

Pigeons, as a minor poultry species, are kept for meat production, racing, and as pets. There is evidence that they may play a role in the transmission of zoonotic AIV to humans without showing severe clinical signs [399]. In pigeons, abundant α2,6-SA with little or no α2,3-SA was found on the epithelium of the respiratory tract, and a similar distribution was found in the intestine, except in the rectum where only α2,3-SA existed [400,401]. 

#### 4.16.8. Emus

After ostrich, the emu is the second-tallest living bird. They are kept for meat, feather, and leather production. Emus are susceptible to AIVs and pandemic influenza viruses [402]. A widespread expression of both α2,3-SA and α2,6-SA receptors was found in the respiratory mucosa of emus, including larynx, trachea, bronchi, and alveoli in lungs. Comparable expression levels of α2,3-SA and α2,6-SA were observed in the ciliated epithelial cells, goblet cells, and non-ciliated epithelial cells, while a higher α2,6-SA expression was detected in the submucosa of the respiratory tract [403]. Moreover, both receptors were detected in the kidneys, cecal tonsils, lymphoid organs, spleen, and cardiac endothelial cells and α2,6-SA was dominant in the epithelial cells of the proventriculus and duodenum [402].

#### 4.16.9. Partridges

Chukar Partridges are gamebirds native to the Middle East and South Asia. Several studies have shown that partridges are less susceptible to infection with AIVs and pandemic influenza virus than, for example, pheasants or quails [395,396]. However, virus excretion was reported for 7 days after infection with some zoonotic and human IAVs [396,404]. Both types of receptors have also been detected in the respiratory tract of red-legged partridges. The olfactory epithelial cells expressed moderate amounts of α2,3-SA and α-2,6-SA. α2,3-SA was expressed mainly in ciliated epithelial cells and less signals were found in respiratory non-ciliated epithelial cells and tracheal ciliated epithelial cells. Likewise, the expression of α2,6-SA receptors was low on the respiratory epithelium, and negative on tracheal epithelial cells. No SA receptors were detected in the epithelial cells in the lungs. Both receptors were also detectable in the duodenum, cecum, and colon [381].

#### 4.16.10. Wild Birds

According to a recent study, there are approximately 9700 bird species worldwide [405]. Wild birds vary in their susceptibility to AIVs. Mallards and gulls are the major reservoir for all AIV subtypes. Human infections can be acquired by visiting LBM, where wild birds are sold, or by hunting, although rarely [68,69]. In 37 wild bird species representing 11 different taxonomic orders, both SA receptors have been detected in the endothelial cells and renal tubular epithelial cells and the endocardium and cardiac endothelial cells [406]. Another study confirmed the existence of human-type receptors in the trachea of several wild birds [401]. 

## 5. Summary and Concluding Remarks

Zoonotic pathogens are responsible for more than 60% of human infectious diseases [407]. Although several zoonotic viruses caused severe human casualties, including the current SARS-CoV2; influenza viruses were responsible for at least four confirmed pandemics in less than a century [408]. IAVs infect a wide range of host species. Avian and swine influenza viruses are of high zoonotic potential, while influenza viruses of bovine, equine, canine, and bat origin are of low zoonotic risk. Animal influenza viruses can transmit directly to humans without intermediate mammal hosts. Beyond pigs, there are several potential mixing vessel hosts for the generation of zoonotic animal influenza viruses, including humans, minks, seals, dogs, cats, zoo animals, camels, and several species of birds. Given the extensive number of wild birds, poultry, swine, and companion animals (dogs and cats) and their close contact to an ever-increasing human population currently standing at eight billion, animal influenza viruses will remain a serious threat for public health. Migratory birds are the highly mobile reservoir for AIVs. Unlike the control of rabies in foxes, there is currently no technology to vaccinate or control IAV infection in the wild-bird reservoir. However, it is possible to limit the infections in domestic reservoirs through improved biosecurity measures, cost-effective culling strategies, and development and use of effective vaccines. Measures are needed to protect non-human mammals (e.g., pigs, minks) from infection with hIAV and AIV and prevent the spread of AIV from and to wild birds. The recent incursion of zoonotic HPAIV H5Nx in wild birds is a game-changer [409]. The virus was transmitted over a long distance by migratory birds from Eurasia via Iceland to the American continent, reaching for the first time, South America [287]. This virus is highly virulent for domestic birds and is able to infect a wide-range of mammals, including humans. Thus, enhanced vigilance is required to monitor the spread and biological alterations of this virus which could develop into a new pandemic pathogen.

## Figures and Tables

**Table 2:** Examples for potential subclinical human infections with AIV H5, H7, and H9 subtypes.

Subtype	Virus/Strain	Total Number of Tested Individuals	Positive Individuals (%)	Country	Year	Reference
H5	H5N1	22	7 (32%)	China	2013–2014	[117]
	H5N1	110	1 (0.9)	China	2006	[118]
	H5N1	87	2 (2.3%)	China	2005–2008	[119]
	H5N1	306	8 (2.6%)	China	2010	[120]
	H5N1	249	5 (2.0%)	China	2010	[121]
	H5N1	501	4 (0.8%)	China	2013	[122]
	H5N1	652	6 (0.9)	China	2014–2016	[112]
	H5N1	2310	18 (0.8%)	China	2014	[123]
	H5N1	964	18 (1.9%)	China	2013–2016	[124]
	H5N1	2124	75 (3.5%)	China	2014–2016	[112]
	H5N1	35159	862 (2.45%)	China	1997–2018	[99] *
	H5N1	2512	9 (0.4%)	South Korea	2003–2004	[125]
	H5N1	200	6 (3%)	Vietnam	2001	[126]
	H5N1	747	37 (5%)	Vietnam	2008–2009	[127]
	H5N1	607	37 (6.1%)	Vietnam	2011	[128]
	H5N1	111	5 (4.5%)	Cambodia	2013	[129]
	H5N1	3594	37 (1.0%)	Cambodia	2006–2014	[130,131,132,133]
	H5N1	800	45 (5.6%)	Thailand	2008	[109]
	H5N1	101	63 (62%)	Indonesia	2012–2014	[111]
	H5N1	376	1 (0.3%)	Turkey	2006	[134]
	H5N1	316	3 (0.9%)	Nigeria	2008–2011	[110]
	H5N1	369	1 (0.3%)	Nigeria	2009	[135]
	H5N1	708	15 (2.1%)	Egypt	2010–2012	[136]
	H5N1	2397	9 (0.4%)	Egypt	2015–2019	[137]
	H5N2	1247	18 (1.4%)	Taiwan	2012	[138]
	H5N2	369	2 (0.5%)	Nigeria	2009	[135]
	H5N2	310	13 (4.2%) §	South Africa	2011–2012	[139]
	H5N2	42	5 (11.9%)	USA	2002–2004	[140]
H7	H7N1	310	6 (1.9%) §	South Africa	2011–2012	[139]
	H7N2	787	34 (4.3%)	USA	2004	[113]
	H7N2	80	1 (1.3%)	USA	2002	[141]
	H7N2	42	6 (14.3%)	USA	2002–2004	[140]
	H7N3	1247	7 (0.6%)	Taiwan	2012	[138]
	H7N3	185	7 (3.8%)	Italy	2003	[142]
	H7N3/H7N1	188	6 (3.2%)	Italy	2008–2010	[143]
	H7N3	157	1 (0.6%)	USA	2009–2010	[144]
	H7N7	1214	1 (0.1)	China	2004	[145]
	H7N7	354	75 (21.2%) §	Pakistan	2010–2011	[146]
	H7N7	490	Not clear	Pakistan	2013	[147]
	H7N7	56	33 (58.9%)	Netherlands	2003	[97]
	H7N7	649	14 (2.2%)	Egypt	2012–2013	[148]
	H7N9	27	1 (3.7%)	China	2013	[149]
	H7N9	96	52 (54.2%)	China	2013	[122]
	H7N9	396	25 (6.3%)	China	2013	[107]
	H7N9	361	3 (0.8%)	China	2013–2014	[150]
	H7N9	10	1 (10%)	China	2013–2014	[117]
	H7N9	12	1 (8.3%)	China	2013–2014	[117]
	H7N9	1056	4 (0.4%)	China	2014	[123]
	H7N9	35	5 (14.3%)	China	2014	[151]
	H7N9	964	9 (0.9%)	China	2013–2016	[124]
	H7N9	225	22 (9.8%)	China	2013–2016	[95]
	H7N9	2124	82 (3.9%)	China	2014–2016	[112]
H9	H9Nx	400	7 (1.8%)	Vietnam	2001	[126]
	H9N2	59590	3313 (5.6%)	China	1990s–2018	[104] *
	H9N2	111	2 (1.8%)	Cambodia	2013	[129]
	H9N2	777	21 (2.7%)	Cambodia	2008	[152]
	H9N2	768	21 (2.7%)	Thailand	2008	[153]
	H9N2	784	40 (5.1%)	Thailand	2008	[153]
	H9N2	338	21 (6.2%)	India	2012	[154]
	H9N2	347	4 (1.2%)	Mongolia	2008–2011	[155]
	H9N2	490	421 (86%)	Pakistan	2013	[147]
	H9N2	332	167 (50.3%)	Pakistan	2016–2017	[156]
	H9N2	354	169 (47.7%) §	Pakistan	2010–2011	[146]
	H9N2	435 §	238 (54.7%)	Pakistan	n.a.	[157]
	H9N2	161	25 (15.5%)	Pakistan	2015–2016	[158]
	H9N2	127	48 (37.7%)	Iran	2006	[159]
	H9N2	182	21 (11.5%)	Iran	2010–2011	[160]
	H9N2	200	20 (10%)	Iran	2012	[161]
	H9N2	34	11 (32.3%)	Lebanon	2005	[162]
	H9N2	363	33 (9.1%)	Romania	2009–2010	[163]
	H9N2	Not mentioned	1	Romania	2010	[164]
	H9N2	682	51 (7.5%)	Egypt	2010–2012	[136]
	H9N2	2397	266 (11.1%)	Egypt	2015–2019	[137]
	H9N2	369	4 (1.1%)	Nigeria	2009	[135]
	H9N2	42	4 (9.5%)	USA	2002–2004	[140]
	H9N2	91	4 (4.4%)	USA	2007–2008	[116]
	H9N2	157	1 (0.6%)	USA	2009–2010	[144]
	H9N2	787	15 (1.9%)	USA	2004	[113]
	H9N8	57	3 (5.3%)	Italy	2005–2006	[114]
Total		~138,730	~6639			

* these references [99,104] are a meta-analysis for >45 studies in China conducted between 1990s and 2018. We did not check all original data in this review. §, this was calculated by the authors from the data provided by the original authors of the indicated studies.

**Table 3:** Confirmed infections of seals with different influenza viruses from 1979 to 2023.

Subtype	Year	Example	Country	Reference
pdmH1N1	2010	A/elephant seal/California/2/2010	USA	[268]
H3N3	1992	A/seal/Massachusetts/3911/1992	USA	[283]
H3N8	2011	A/harbor seal/New Hampshire/179629/2011	USA	[282]
H3N8	2011	A/harbor seal/Massachusetts/1/2011	USA	[279]
H3N8	2017	A/grey seal/England/027661/2017	UK	[284]
H4N5	1982	A/seal/Massachusetts/133/1982	USA	[279]
H4N6	2002	A/Caspian seal/Russia/1884/2002	Russia	[285]
H4N6	2012	A/Caspian seal/Russia/T1/2012	Russia	[286]
H5N1	2022	A/harbor seal/Maine/22-020455-001-original/2022	USA	[275]
H5N1	2022	Harbor seal	Canada	[287]
H5N1	2022–2023	Grey and harbor seals	UK	[288,289]
H5N8	2016	A/grey seal/361-10/BalticPL/2016	Poland	[290]
H5N8	2020	A/seal/England/AVP-031141/2020	UK	[291]
H5N8	2021	A/seal/Germany-SH/AI05373/2021	Germany	[292]
H5N8	2021	A/seal/Sweden/2021	Sweden	[292]
H5N8	2021	A/harbor seal/Denmark/521-2/2021	Denmark	[287]
H7N7	1980	A/seal/Massachusetts/1/1980	USA	[276,293]
H10N7	2021	A/harbor seal/British Colombia/OTH-52-1/2021	Canada	[274]
H10N7	2014	A/harbor seal/Germany/1/2014	Europe *	[272]

pdmH1N1 = pandemic H1N1 in 2009, * = Germany, Sweden, Netherlands, and Denmark

## Data Availability

Data are contained within the article.
